# PKACs attenuate innate antiviral response by phosphorylating VISA and priming it for MARCH5-mediated degradation

**DOI:** 10.1371/journal.ppat.1006648

**Published:** 2017-09-21

**Authors:** Bing-Ru Yan, Lu Zhou, Ming-Ming Hu, Mi Li, Heng Lin, Yan Yang, Yan-Yi Wang, Hong-Bing Shu

**Affiliations:** 1 College of Life Sciences, Wuhan University, Wuhan, China; 2 Medical Research Institute, School of Medicine, Wuhan University, Wuhan, China; 3 Wuhan Institute of Virology, State Key Laboratory of Virology, Chinese Academy of Sciences, Wuhan, China; Institute of Biochemistry and Cell Biology, CHINA

## Abstract

Sensing of viral RNA by RIG-I-like receptors initiates innate antiviral response, which is mediated by the central adaptor VISA. How the RIG-I-VISA-mediated antiviral response is terminated at the late phase of infection is enigmatic. Here we identified the protein kinase A catalytic (PKAC) subunits α and β as negative regulators of RNA virus-triggered signaling in a redundant manner. Viral infection up-regulated cellular cAMP levels and activated PKACs, which then phosphorylated VISA at T54. This phosphorylation abrogated virus-induced aggregation of VISA and primed it for K48-linked polyubiquitination and degradation by the E3 ligase MARCH5, leading to attenuation of virus-triggered induction of downstream antiviral genes. PKACs-deficiency or inactivation by the inhibitor H89 potentiated innate immunity to RNA viruses in cells and mice. Our findings reveal a critical mechanism of attenuating innate immune response to avoid host damage at the late phase of viral infection by the house-keeping PKA kinase.

## Author summary

VISA is a central adaptor protein required for innate immune response to RNA virus. Phosphorylation of VISA by protein kinase A leads to its polyubiquitination and degradation by the E3 ligase MARCH5 at the late phase of viral infection, which provides a critical control mechanism for the host to avoid excessive and harmful immune response.

## Introduction

Innate immune response is the first line of host defense against invading microbial pathogens. The structurally conserved components of microbes called pathogen-associated molecular patterns (PAMPs) are recognized by host pattern-recognition receptors (PRRs), which initiates signaling pathways that lead to induction of type I interferons (IFNs), proinflammatory cytokines and other downstream effector genes [1]. During RNA virus infection, viral RNAs, including the invading viral RNAs and RNA intermediates produced during viral replication, act as PAMPs that are mostly recognized by the cytoplasmic RIG-I-like receptor (RLR) family members including RIG-I and MDA5 [2]. Although RIG-I and MDA5 sense distinct types of viral RNAs, they utilize a common adaptor protein called VISA (also known as MAVS, IPS-1 or Cardif) to transmit signals [3–6]. Upon binding to viral RNAs, RLRs are recruited to VISA located on the mitochondrial outer membrane, and this induces aggregation and activation of VISA [7]. VISA then acts as a central platform for recruitment of downstream signaling components, including TRAF2/3/5/6, cIAP1/2 and WDR5 [6, 8–10]. In these complexes, TRAF6 functions redundantly with TRAF2 and TRAF5 to activate IRF3 and IKK [11]. These processes lead to eventual transcription of downstream antiviral genes, including type I IFNs, proinflammatory cytokines and other effectors [12, 13].

Protein phosphorylation and dephosphorylation play important roles in innate immune responses to RNA viruses by regulating the activation and deactivation of multiple RLR-mediated signaling components, such as RIG-I, VISA, TRAF3, TBK1 and IRF3 [14–18]. In some cases, the enzymes that are responsible for their modifications are unknown. The RLR-mediated signaling pathways are also heavily regulated by other post-translational modifications, such as ubiquitination, sumoylation, methylation and [19–23]. How post-translational modifications cross-talk to regulate innate antiviral response remains enigmatic.

Protein kinase A (PKA) is one of the first identified protein kinases, which is critically important for many divergent cellular processes, such as metabolism, cell cycle, cell migration, differentiation and apoptosis. PKA exists as a tetrameric holoenzyme with two regulatory subunits and two catalytic subunits in its inactive form. Cyclic adenylyl monophosphate (cAMP) causes dissociation of the inactive holoenzyme into a dimer of regulatory subunits bound to four cAMP and two free monomeric catalytic subunits [24]. Four regulatory subunits (PKARIα, PKARIβ, PKARIIα and PKARIIβ) and three catalytic subunits (PKACα, PKACβ and PKACγ) have been identified in humans. PKACα and PKACβ are ubiquitously expressed in most examined tissues, but PKACγ is specifically expressed in testis. Human PKACα and PKACβ are highly homologous, which share ~93% sequence identity at the amino acid level [25].

In this report, we identified PKACα and PKACβ as two redundant negative regulators of RNA virus-triggered induction of downstream antiviral genes. Viral infection activated PKACs, which in turn phosphorylated VISA at T54, leading to impairment of VISA aggregation and its K48-linked polyubiquitination and degradation by the E3 ligase MARCH5. We also showed that PKACs-deficiency or inactivation potentiated innate immunity to RNA viruses in cells and mice. Our findings reveal a critical mechanism of attenuating innate immune response at the late phase of viral infection and establish an un-described function for PKA in innate antiviral response.

## Results

### PKACα and PKACβ negatively regulate VISA-mediated signaling

**Fig 1:**
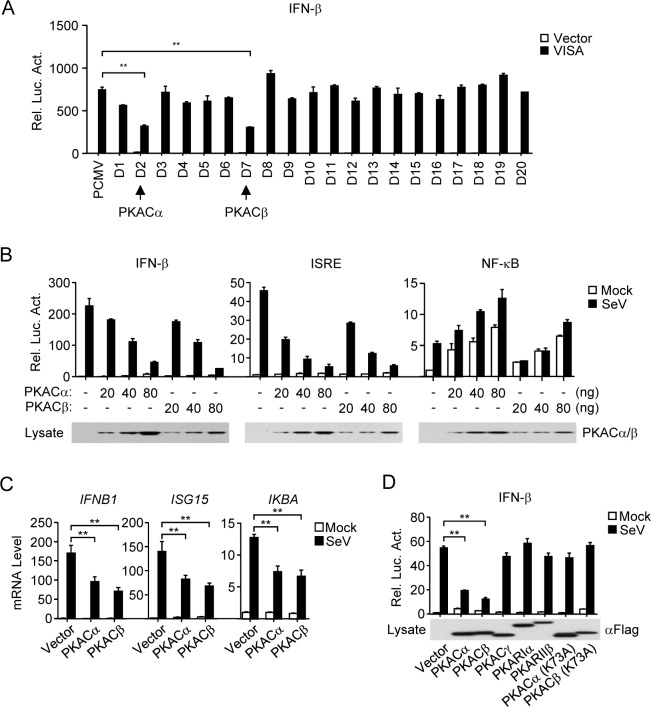
PKACα or PKACβ inhibits SeV-induced and VISA-mediated signaling. (A) Screening of kinase cDNA clones that inhibit VISA-mediated activation of the IFN-β promoter. HEK293 cells were transfected with the IFN-β promoter luciferase plasmid, mammalian expression plasmids for VISA and the kinase cDNA clones for 20 h before luciferase assays were performed. Representative data is shown. (B) PKACα and PKACβ inhibit SeV-triggered activation of the IFN-β promoter and ISRE but not NF-κB. HEK293 cells were transfected with the indicated reporter plasmids and increased amounts of Flag-PKACα/β plasmids for 20 h, then infected with SeV (MOI = 1) or left untreated for 12 h before luciferase assays were performed. The lower blots show the expression levels of transfected PKACα/β as detected by anti-Flag antibody. (C) PKACα and PKACβ inhibit SeV-triggered induction of downstream antiviral genes. HEK293 cells were transfected with the indicated expression plasmids for 20 h, then infected with SeV (MOI = 1) or left untreated for 8 h before qPCR was performed. (D) Effects of PKA subunits and mutants on SeV-triggered activation of the IFN-β promoter. HEK293 cells were transfected with the IFN-β promoter luciferase plasmid and the indicated Flag-tagged expression plasmids for 20 h, then infected with SeV (MOI = 1) or left untreated for 12 h before luciferase assays were performed. The lower blot shows the expression levels of the transfected proteins.

VISA is a central adaptor protein in innate immune response to RNA virus. To identify potential kinases that regulate VISA-mediated signaling, we screened a cDNA library contains 352 kinase clones. We found that PKACα and PKACβ markedly inhibited VISA-mediated activation of the IFN-β promotor in HEK293 cells (Fig 1, panel A). PKACα and PKACβ also dose-dependently inhibited Sendai virus (SeV)-induced activation of the IFN-β promoter and ISRE, an enhancer motif for activated IRF3. Overexpression of PKACα and PKACβ activated NF-κB (Fig 1, panel B), which is consistent with previous reports that PKACα and PKACβ can phosphorylate p65 on S276 [26, 27]. Overexpression of PKACα and PKACβ also inhibited SeV-induced transcription of downstream genes such as *IFNB1*, *ISG15* and *IKBA* (Fig 1, panel C). In contrast, the testis specific PKA catalytic subunit PKACγ, the PKA regulatory subunits PKARIα and PKARIIβ, or the catalytic inactive mutants of PKACα (K73A) and PKACβ (K73A) [28], did not inhibit SeV-induced activation of the IFN-β promoter (Fig 1, panel D). These results suggest that PKACα and PKACβ can inhibit RNA virus-triggered and VISA-mediated induction of downstream antiviral genes.

**Fig 2:**
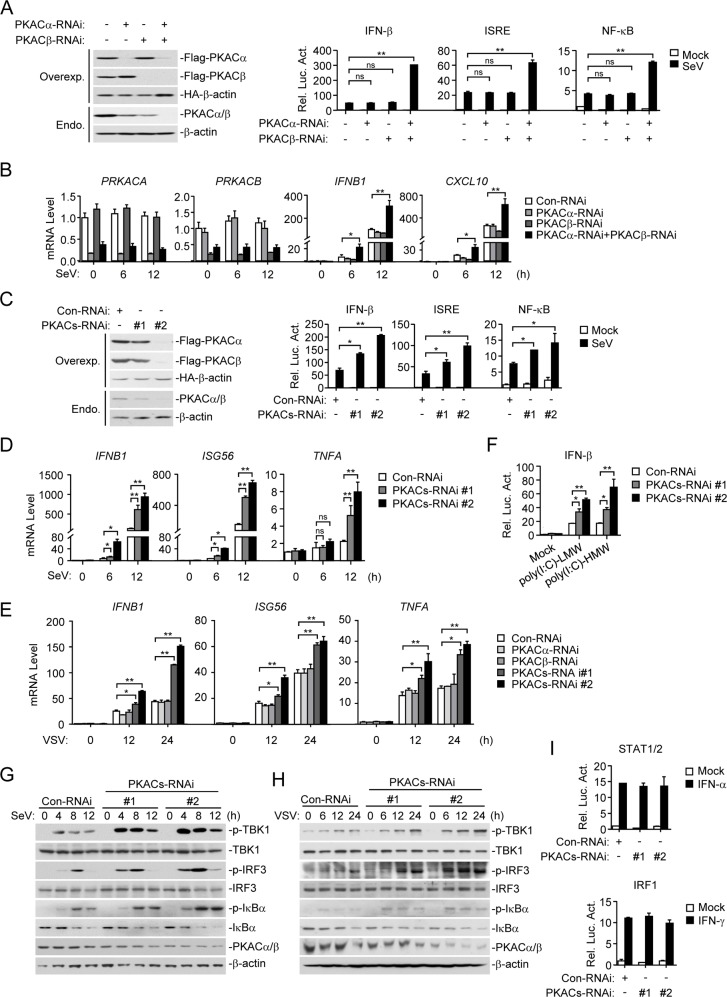
Simultaneous knockdown of PKACα and PKACβ potentiates RNA virus-triggered signaling. (A) Left panels: Knockdown efficiencies of PKACa and PKACb by RNAi. For the upper three panels, HEK293 cells were transfected with Flag-PKACα/β, HA-β-actin, and the indicated RNAi plasmids for 36 h, and then analyzed by immunoblots with anti-Flag or anti-HA antibodies. For the lower two panels, HEK293 cells were transfected with the indicated RNAi plasmids for 48 h before immunoblot analysis with the indicated antibodies. Right histographs: Effects of PKACα and PKACβ knockdown on SeV-induced signaling. HEK293 cells were transfected with the indicated reporter and RNAi plasmids for 36 h, then infected with SeV (MOI = 1) or left untreated for 12 h before luciferase assays were performed. (B) Effects of PKACα and PKACβ knockdown on SeV-induced transcription of downstream genes. HEK293 cells were transfected with the indicated RNAi plasmids for 12 h. The cells were then selected with puromycin for 24 h and then infected with SeV (MOI = 1) for the indicated times before qPCR was performed. (C) Effects of simultaneous knockdown of PKACα and PKACβ on SeV-induced signaling. The experiments were similarly performed as in A by using RNAi plasmids simultaneous target PKACα and PKACβ. (D) Effects of simultaneous knockdown of PKACα and PKACβ on SeV-induced transcription of downstream genes. The experiments were similarly performed as in B by using RNAi plasmids simultaneous target PKACα and PKACβ. (E) Effects of knockdown of PKACα and PKACβ on VSV-induced transcription of downstream genes. THP1 cells were transfected with the indicated siRNA for 36 h and then infected with VSV (MOI = 1) for the indicated times before qPCR was performed. (F) Knockdown of PKACs potentiates cytoplasmic poly(I:C)-triggered activation of the IFN-β promoter. HEK293 cells were transfected with the IFN-β promoter luciferase plasmid and the indicated RNAi plasmids for 36 h, then transfected with low molecular weight (LMW) or high molecular weight (HMW) poly(I:C) for 12 h before luciferase assays were performed. (G) Knockdown of PKACs inhibits SeV-induced phosphorylation of TBK1, IRF3 and IκBα. HEK293 cells were transfected with the indicated RNAi plasmids and selected with puromycin, then infected with SeV (MOI = 1) for the indicated times. Cell lysates were analyzed by immunoblots with the indicated antibodies. (H) Knockdown of PKACs inhibits VSV-induced phosphorylation of TBK1, IRF3 and IκBα. THP1 cells were transfected with the indicated siRNA for 36 h and then infected with VSV (MOI = 1) for the indicated times. Cell lysates were analyzed by immunoblots with the indicated antibodies. (I) Effects of PKACs knockdown on IFN-α-induced STAT1/2 and IFN-γ-induced IRF1 promoter activation. HEK293 cells were transfected with the indicated reporter and PKACs RNAi plasmids for 36 h, then treated with the indicated cytokines or left untreated for 12 h before luciferase assays were performed.

We next determined whether endogenous PKACα and PKACβ are involved in regulation of virus-induced signaling. We found that knockdown of either PKACα or PKACβ by RNAi had no marked effects on SeV-induced activation of the IFN-β promoter, ISRE and NF-κB in reporter assays. However, simultaneous knockdown of both PKACα and PKACβ markedly potentiated SeV-induced activation of the IFN-β promoter, ISRE and NF-κB (Fig 2, panel A). Consistently, simultaneous but not individual knockdown of PKACα and PKACβ potentiated SeV-induced transcription of *IFNB1* and *CXCL10* genes (Fig 2, panel B).

Because PKACα and PKACβ are highly conserved at both amino acid and mRNA sequence levels, we constructed two more RNAi plasmids (PKACs-RNAi #1 and #2), each of them simultaneously target both of human PKACα and PKACβ mRNAs. Simultaneous knockdown of the two catalytic subunits PKACα and PKACβ (referred below as PKACs) by these two RNAi plasmids potentiated SeV-induced activation of the IFN-β promoter, ISRE and NF-κB (Fig 2, panel C), as well as SeV or vesicular stomatitis virus (VSV)-induced transcription of *IFNB1*, *ISG56* and *TNFA* genes (Fig 2, panel D&E). The degrees of potentiation were correlated to the knockdown efficiencies of the RNAi plasmids (Fig 2, panel C, D&E). Knockdown of PKACs also potentiated IFN-β promoter activation triggered by poly(I:C) transfected into HEK293 cells (Fig 2, panel F). In addition, knockdown of PKACs also markedly enhanced SeV or VSV-induced phosphorylation of TBK1, IRF3 and IκBα (Fig 2, panel G&H). However, knockdown of PKACs had no marked effects on IFN-α-induced activation of STAT1/2 and IFN-γ-induced activation of the IRF1 promoter (Fig 2, panel I). These data suggest that PKACα and PKACβ negatively regulate RNA virus-induced expression of downstream genes in a redundant manner.

### Viral infection induces PKACs activation

**Fig 3:**
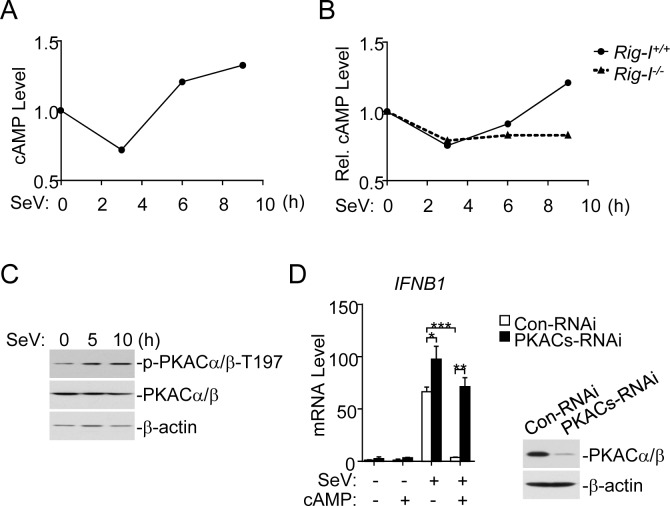
Viral infection induces cAMP and PKACs activation. (A) Levels of cellular cAMP following SeV infection. HeLa cells were infected with SeV (MOI = 1) for the indicated times. Cell lysates were analyzed by cAMP enzyme immunoassay kit. (B) Levels of cellular cAMP following SeV infection in *Rig-I*^+/+^ and *Rig-I*^-/-^ MEFs. The indicated MEFs were infected with SeV (MOI = 1) for the indicated times. Cell lysates were analyzed by cAMP enzyme immunoassay kit. (C) SeV infection induces PKACs phosphorylation. HeLa cells were infected with SeV (MOI = 1) for the indicated times before immunoblot analysis with the indicated antibodies. (D) Effects of exogenous cAMP on SeV-induced transcription of *IFNB1* gene. HeLa cells were transfected with PKACs RNAi plasmid and selected with puromycin, then infected with SeV (MOI = 1) and treated with cAMP (1x10^7^ pmol/mL) or left untreated for 6 h before qPCR was performed. The right blots show the expression levels of PKACα/β.

To investigate how PKACs function following viral infection, we determined whether viral infection triggers the accumulation of cellular cAMP. We found that SeV infection caused a transient decrease of cellular cAMP level at the early phase of infection (3 h) but marked increase at the late phase of infection (Fig 3, panel A). The cAMP levels in RIG-I-deficient cells at the late phase of infection were not increased (Fig 3, panel B), suggesting that the increase of cAMP level at the late phase of viral infection was dependent on RIG-I-mediated signaling. In addition, SeV infection induced the phosphorylation of PKACs on T197 (Fig 3, panel C), which is a hallmark of PKACs activation [29, 30]. These results suggest that viral infection leads to increase of cellular cAMP levels and activation of PKACs at the late phase of infection in a RIG-I-dependent manner.

Additionally, we determined the effects of exogenous cAMP on virus-induced expression of downstream antiviral genes. We found that introduction of exogenous cAMP into the cells abolished SeV-induced transcription of *IFNB1* gene, and knockdown of PKACs reversed the effects of exogenous cAMP (Fig 3, panel D). These results suggest that viral infection induced increase of cAMP levels and activation of PKACs, which in turn inhibit virus-triggered induction of downstream antiviral genes in a negative feed-back manner.

### PKACs catalyze phosphorylation of VISA at T54

**Fig 4:**
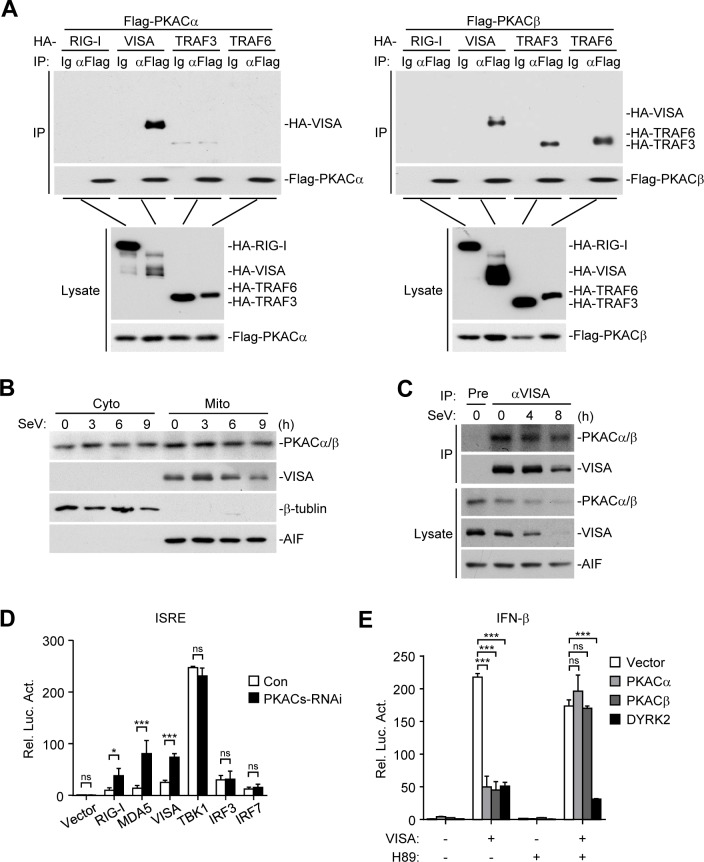
PKACs target VISA in the virus-triggered pathways. (A) VISA interacts with PKACα and PKACβ. HEK293 cells were transfected with the indicated plasmids for 20 h before co-immunoprecipitation and immunoblot analysis with the indicated antibodies. (B) Distribution of PKACs. HEK293 cells were infected with SeV (MOI = 1) for the indicated times and then fractionated the subcellular fractions were equilibrated to equal volumes and analyzed by immunoblots with the indicated antibodies. (C) Endogenous VISA is associated with PKACs in the mitochondria. HEK293 cells were infected with SeV (MOI = 1) for the indicated times. The mitochondria were isolated by cell fractionation and the mitochondrial lysates were subjected to immunoprecipitation and immunoblot analysis with the indicated antibodies. (D) Effects of PKACs knockdown on ISRE activation by various components. HEK293 cells were transfected with ISRE luciferase and PKACs RNAi plasmids for 36 h, then transfected with the indicated expression plasmids for 20 h before luciferase assays were performed. (E) Effects of H89 on PKACs-mediated inhibition of VISA activity. HEK293 cells were transfected with the IFN-β promoter luciferase and the indicated expression plasmids for 20 h, then transfected with VISA plasmid for 20 h and treated with H89 (10 μg/mL) or left untreated for 8 h before luciferase assays were performed.

**Fig 5:**
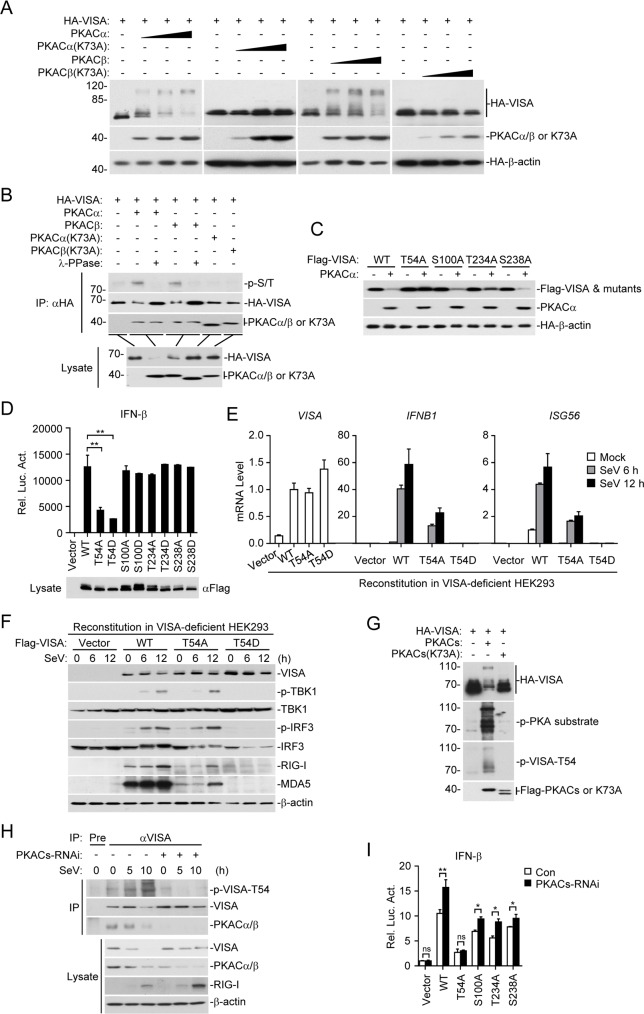
PKACs phosphorylate VISA at T54. (A) Effects of PKACs and their kinase inactive mutants on VISA modification. HEK293 cells were transfected with HA-VISA and increased amounts of the indicated expression plasmids for 20 h before immunoblot analysis with the indicated antibodies. (B) VISA is serine/threonine phosphorylated by PKACs. HEK293 cells were transfected with HA-VISA and the indicated expression plasmids for 20 h. Cell lysates were immunoprecipitated with anti-HA, and the immunoprecipitates were treated with buffer or lambda protein phosphatase (λ-PPase) and then analyzed by immunoblots with the indicated antibodies. (C) Effects of PKACα on VISA and its mutants. HEK293 cells were transfected with the indicated expression plasmids for 20 h and then analyzed by immunoblots with the indicated antibodies. (D) Effects of VISA and its mutants on IFN-β promoter activation. HEK293 cells were transfected with the IFN-β promoter luciferase and Flag-VISA or its mutants plasmids for 20 h before luciferase assays were performed. The blot shows the expression levels of the transfected VISA and its mutants. (E) Effects of VISA and its mutants on SeV-induced transcription of *IFNB1* gene. VISA-deficient HEK293 cells reconstituted with VISA or its mutants were infected with SeV (MOI = 1) for the indicated times before qPCR analysis. (F) Effects of VISA and its mutants on SeV-induced TBK1 and IRF3 phosphorylation. VISA-deficient HEK293 cells reconstituted with VISA or its mutants were infected with SeV (MOI = 1) for the indicated times before immunoblot analysis with the indicated antibodies. (G) PKACs phosphorylate VISA at T54. HEK293 cells were transfected with HA-VISA, Flag-tagged PKACα and Flag-PKACβ and their mutants for 20 h. Cell lysates were analyzed by immunoblots with the indicated antibodies. (H) Endogenous VISA is phosphorylated at T54 by PKACs after SeV infection. HEK293 cells were transfected with the indicated plasmids and selected with puromycin, then infected with SeV (MOI = 1) for the indicated times. Cell lysates were subjected to immunoprecipitation and immunoblot analysis with the indicated antibodies. (I) Effects of PKACs knockdown on activation of the IFN-β promoter by VISA and its mutants. HEK293 cells were transfected with the IFN-β promoter luciferase and PKACs RNAi plasmid for 36 h, then transfected with Flag-VISA or its mutants for 20 h before luciferase assays were performed.

We next determined the molecular mechanisms responsible for the inhibitory effects of PKACs on virus-triggered induction of downstream genes. In transient transfection and co-immunoprecipitation experiments, PKACα interacted with VISA, while PKACβ interacted with VISA, TRAF3 and TRAF6. Neither PKACα nor PKACβ interacted with RIG-I (Fig 4, panel A). Cellular fractionation experiments indicated that PKACs were localized in the cytosol and at the mitochondria (Fig 4, panel B). Endogenous co-immunoprecipitation experiments indicated that PKACs were constitutively associated with VISA before and after SeV infection (Fig 4, panel C). In reporter assays, knockdown of PKACs enhanced upstream components RIG-I-, MDA- and VISA- but not downstream components TBK1-, IRF3- or IRF7-mediated ISRE activation (Fig 4, panel D). H89 is a specific and potent PKA inhibitor [31], which completely reversed the inhibitory effects of PKACs on VISA-mediated activation of the IFN-β promoter (Fig 4, panel E). In the same experiments, H89 had no effects on the inhibition of VISA-mediated activation of the IFN-β promoter by DYRK2, a kinase that negatively regulates virus-triggered signaling by targeting TBK1 for phosphorylation [16]. Collectively, these results suggest that PKACs inhibit virus-triggered induction of downstream antiviral genes by targeting VISA. Consistently, overexpression of PKACα or PKACβ but not their kinase inactive mutants caused a shift of VISA to higher molecular weight species (Fig 5, panel A). These higher molecular weight species of VISA were recognized by an antibody to phosphorylated serine and/or threonine (p-S/T) and removed by treatment with lambda protein phosphatase (λ-PPase) (Fig 5, panel B). These results suggest that PKACs phosphorylate VISA.

We next determined the residues of VISA that are phosphorylated by PKACs. Prediction by GPS3.0 program indicates that VISA contains four consensus PKA phosphorylation residues, including T54, S100, T234 and S238. Among the phosphorylation sites, T54 is highly conserved in mammals and the only residue located in the N-terminal CARD-like domain of VISA. Mutagenesis indicated that PKACα phosphorylated wild-type VISA and the VISA mutants VISA(S100A), VISA(T234A) and VISA(S238A) but not VISA(T54A) (Fig 5, panel C). Reporter assays indicated that mutation of S100, T234 and S238 of VISA to either alanine (A) or aspartic acid (D) had no marked effects on its ability to activate the IFN-β promoter. However, mutation of T54 of VISA to D, which mimics its phosphorylation, dramatically impaired its ability to activate downstream signaling. Unexpectedly, mutation of T54 of VISA to A, which mimics its un-phosphorylated form, also impaired its activity, though to a lesser degree (Fig 5, panel D). We further investigated the functions of the T54 mutants of VISA by reconstituting them into VISA-deficient HEK293 cells. We found that VISA(T54D) completely lost the ability to mediate SeV-triggered induction of downstream *IFNB1* and *ISG56* genes, while VISA(T54A) partially maintained the ability in comparison to wild-type VISA (Fig 5, panel E). Consistently, SeV-induced phosphorylation of TBK1 and IRF3 was partially and completely impaired in VISA(T54A)- and VISA(T54D)-reconstituted cells respectively in comparison to wild-type VISA-reconstituted cells (Fig 5, panel F). These results suggest that T54 is probably the target residue of PKACs.

To determine whether PKACs indeed target T54 of VISA for phosphorylation, we generated a rabbit polyclonal antibody to a peptide containing phosphorylated T54 (p-VISA-T54). Immunoblot analysis indicated that PKACs but not their kinase inactive mutants caused phosphorylation of VISA at T54 (Fig 5, panel G). The phosphorylation of VISA at T54 was dramatically enhanced following SeV infection for 10 hours, while knockdown of PKACs impaired the phosphorylation of VISA at T54 and increased induction of RIG-I (Fig 5, panel H). In addition, knockdown of PKACs potentiated wild-type VISA and its S100A, T234A and S238A mutants but not T54A mutant mediated activation of the IFN-β promoter (Fig 5, panel I). Taken together, these results suggest that PKACs inhibit SeV-triggered induction of downstream genes by direct phosphorylation of VISA at T54.

### PKACs impair VISA aggregation and promote its degradation by MARCH5

**Fig 6:**
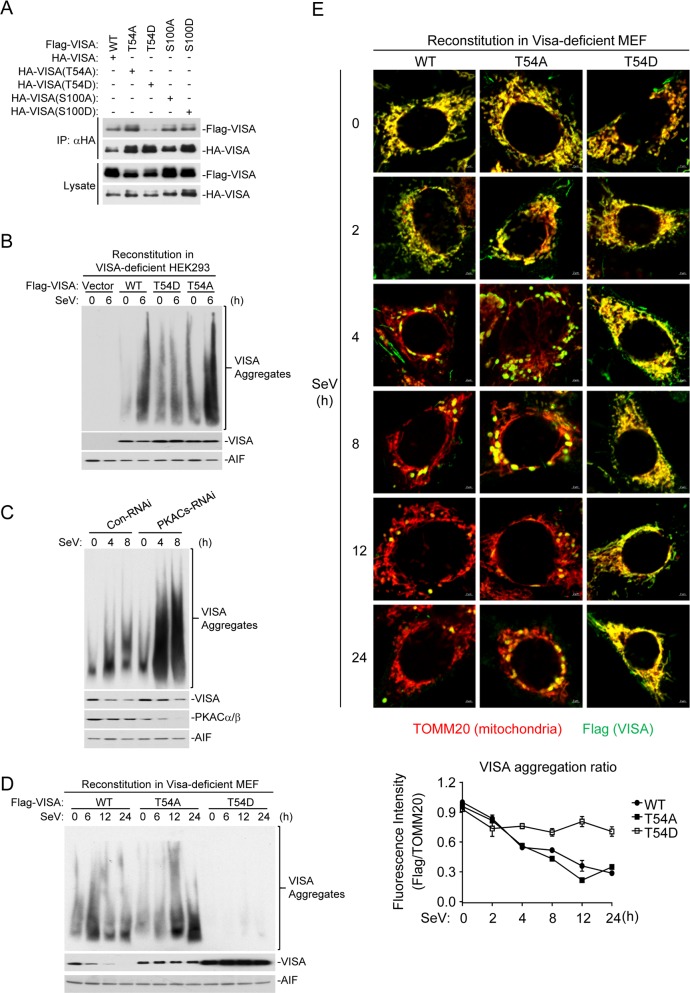
PKACs impair VISA aggregation. (A) Oligomerization of VISA and its mutants. HEK293 cells were transfected with the indicated expression plasmids for 20 h before co-immunoprecipitation and immunoblot analysis with the indicated antibodies. (B) Effects of T54 mutation on SeV-induced aggregation of VISA in the mitochondria. Crude mitochondrial extracts were prepared from HEK293 cells reconstituted with VISA and its mutants and infected with SeV (MOI = 1) for the indicated times. The extracts were fractionated by SDD-AGE and SDS-PAGE and analyzed by immunoblots with the indicated antibodies. (C) Effects of PKACs knockdown on SeV-induced VISA aggregation. Crude mitochondrial extracts were prepared from control or PKACs-RNAi stable-transduced HEK293 cells infected with SeV (MOI = 1) for the indicated times. The extracts were fractionated by SDD-AGE and SDS-PAGE and analyzed by immunoblots with the indicated antibodies. (D) Effects of T54 mutation on SeV-induced aggregation of VISA in MEF cells. Crude mitochondrial extracts were prepared from MEF cells reconstituted with VISA and its mutants and infected with SeV (MOI = 1) for the indicated times. The extracts were fractionated by SDD-AGE and SDS-PAGE and analyzed by immunoblots with the indicated antibodies. (E) Confocal microscopy for virus-induced aggregation of VISA and its mutants. Flag-tagged VISA or its mutants were reconstituted into *Visa*^*-/-*^ MEFs. The cells were infected with SeV (MOI = 1) for the indicated times before confocal microscopy. The images are representative of > 50% of the cells under examination.

It has been demonstrated that VISA forms prion-like aggregates on the mitochondrial membrane to activate innate immune response after viral infection [7]. We next investigated whether phosphorylation of VISA at T54 by PKACs impairs the formation of VISA aggregates. Co-immunoprecipitation experiments indicated that the self-association of VISA(T54D) was markedly decreased compared to wild-type VISA, VISA(T54A), VISA(S100A), or VISA(S100D) (Fig 6, panel A). Reconstitution experiments indicated that SeV-induced aggregation of VISA(T54D) was decreased in comparison to wild-type VISA and VISA(T54A) (Fig 6, panel B). In addition, knockdown of PKACs increased the formation of VISA aggregates following SeV infection (Fig 6, panel C). Confocal microscopy indicated that VISA(T54A) formed aggregates more dramatically than wild-type VISA at the early phase of infection (2–8 h) and some aggregates remained even at the late phase of infection (24 h). Interestingly, VISA(T54D) colocalized with mitochondria but did not form aggregates before and after viral infection. In addition, VISA(T54D) was also not degraded after viral infection (Fig 6, panel D&E). These results suggest that PKACs-mediated phosphorylation of VISA at T54 impairs its aggregation and activation.

**Fig 7:**
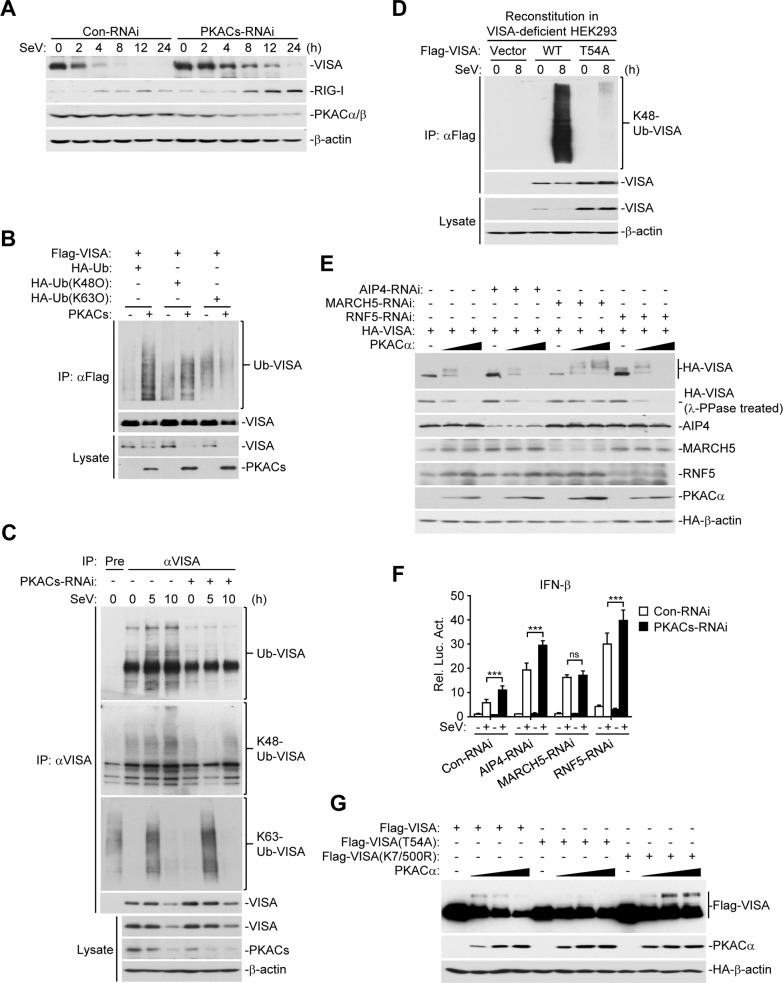
PKACs promote VISA degradation by MARCH5. (A) Knockdown of PKACs inhibits SeV-induced VISA degradation. HEK293 cells were transfected with the indicated RNAi plasmids and selected with puromycin, then infected with SeV (MOI = 1) for the indicated times. Cell lysates were analyzed by immunoblots with the indicated antibodies. (B) PKACs promotes K48- but not K63-linked polyubiquitination of VISA. HEK293 cells were transfected with the indicated plasmids for 20 h, followed by co-immunoprecipitation and immunoblotting analysis. (C) Knockdown of PKACs inhibits SeV-induced K48-linked polyubiquitination of VISA. PKACs-RNAi stable-transduced HEK293 cells were infected with SeV (MOI = 1) for the indicated times before co-immunoprecipitation and immunoblotting analysis. (D) Mutation of T54 of VISA to alanine impairs its K48-linked polyubiquitination induced by SeV. VISA-deficient HEK293 cells reconstituted with VISA or its mutants were infected with SeV (MOI = 1) for the indicated times before co-immunoprecipitation and immunoblot analysis. (E) Effects of knockdown of AIP4, MARCH5 or RNF5 on PKACα-induced phosphorylation and degradation of VISA. HEK293 cells were transfected with the indicated RNAi plasmids and selected with puromycin, then transfected with HA-VISA and increased amounts of Flag-PKACα for 20 h before immunoblot analysis with the indicated antibodies. For the second blot from the top, the same samples were treated with λ-PPase before immunoblot analysis. (F) Effects of knockdown of PKACs on activation of the IFN-β promoter triggered by SeV and knockdown of three E3 ligases. HEK293 cells were transfected with the IFN-β promoter luciferase and the indicated RNAi plasmids for 48 h before luciferase assays were performed. (G) Effects of PKACα on the levels of VISA and its mutants. HEK293 cells were transfected with Flag-VISA or its mutants and increased amounts of Flag- PKACα plasmid for 20 h before immunoblot analysis with the indicated antibodies.

In our experiments, we routinely found that PKACα and PKACβ but not their kinase inactive mutants caused down-regulation of VISA (Fig 5, panel A), while knockdown of PKACs up-regulated the levels of endogenous VISA (Fig 5, panel H). Kinetic experiments indicated that the levels of VISA were gradually down-regulated from 2–24 h after viral infection, and knockdown of PKACs slowed SeV-triggered down-regulation of VISA (Fig 7, panel A). We therefore tested the hypothesis that phosphorylation of VISA at T54 by PKACs impairs its aggregation and primes it for polyubiquitination and proteasomal degradation. We found that overexpression of PKACs enhanced K48- but not K63-linked polyubiquitination of VISA (Fig 7, panel B). Conversely, knockdown of PKACs inhibited SeV-induced K48- but not K63-linked polyubiquitination of VISA (Fig 7, panel C). Reconstitution experiments indicated that SeV-induced K48-linked polyubiquitination and degradation of VISA(T54A) was impaired in comparison to wild-type VISA (Fig 7, panel D). These results suggest that phosphorylation of VISA at T54 by PKACs primes it for K48-linked polyubiquitination and degradation.

Previous studies have identified AIP4, MARCH5 and RNF5 as E3 ubiquitin ligases that catalyze K48-linked polyubiquitination of VISA [32–34]. We found that individually knockdown of the examined three E3 ligases did not affect the phosphorylation of VISA by PKACα. However, knockdown of MARCH5 but not RNF5 or AIP4 dramatically inhibited the degradation of VISA mediated by PKACα (Fig 7, panel E). In addition, knockdown of MARCH5 but not RNF5 and AIP4 inhibited the synergistic activation of the IFN-β promoter induced by SeV and PKACs knockdown (Fig 7, panel F). Previously, it has been demonstrated that MARCH5 targets K7 and K500 of VISA for K48-linked polyubiquitination and degradation [33]. We found that PKACα caused degradation of wild-type VISA but not VISA(T54A) or VISA(K7/500R), in which either the PKACs-mediated phosphorylation or MARCH5-mediated K48-linked polyubiquitination residues are mutated (Fig 7, panel G). Collectively, these results suggest that PKACs-mediated phosphorylation of VISA at T54 primes it for K48-linked polyubiquitination and degradation by MARCH5.

### PKACs inhibit antiviral response *in vivo*

**Fig 8:**
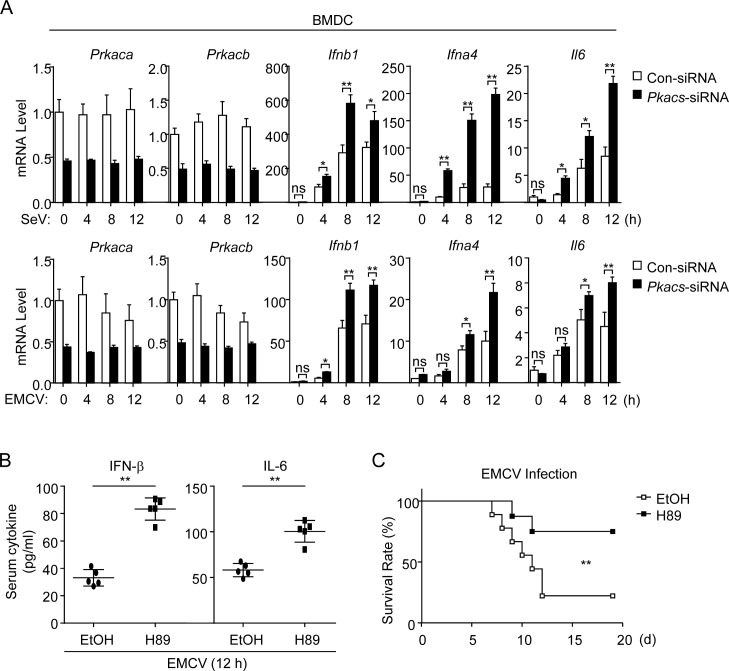
PKA negatively regulates innate antiviral response in mice. (A) Knockdown of PKACs inhibits SeV- and EMCV-induced transcription of downstream antiviral genes in BMDCs. BMDCs were transfected with PKACs siRNA for 36 h, then infected with SeV (MOI = 1) or EMCV (MOI = 1) for the indicated times before qPCR analysis. (B) H89 potentiates EMCV-induced serum cytokine levels in mice. Mice (n = 6) were infected i.p. with EMCV at 1×10^7^ pfu per mouse for 12 h before serum cytokines were measured by ELISA. (C) H89 inhibits EMCV-induced death in mice. Mice (n = 8) were infected i.p. with EMCV at 1×10^5^ pfu per mouse, and the survival rates of mice were observed and recorded for 2 weeks.

Finally, we investigated whether PKACs regulate innate antiviral response in immune cells and *in vivo*. We found that induction of *Ifnb1*, *Ifna4* and *Il6* mRNAs by either SeV or encephalomyocarditis virus (EMCV) in bone marrow-derived dendritic cells (BMDCs) was markedly increased by knockdown of PKACs (Fig 8, panel A). In addition, mice treated with the PKA inhibitor H89 produced higher levels of serum cytokines including IFN-α4, IFN-β and IL-6 upon EMCV infection (Fig 8, panel B) and were more resistant to EMCV-induced death (Fig 8, panel C). These results suggest that PKA negatively regulates innate immune responses to RNA viruses in mice.

## Discussion

Proper and efficient innate immune response at the early phase of infection is critical for clearance of viruses, while timely termination of innate antiviral response at the late phase of infection is important for avoiding harmful immune damage and death of the host. How the innate antiviral response is delicately regulated has been heavily investigated in the past decade. In this report, we found that the house-keeping kinase PKA played an essential role in attenuating innate immune response to RNA virus by inactivating the central adaptor protein VISA in the virus-triggered signaling pathways.

Overexpression of PKACα and PKACβ, but not PKACγ, the PKA regulatory subunits PKARIα and PKARIIβ, or the catalytic inactive mutants of PKACα (K73A) and PKACβ (K73A) markedly inhibited SeV-triggered and VISA-mediated induction of downstream antiviral genes. Interestingly, knockdown of either PKACα or PKACβ had no marked effects on SeV-triggered signaling, but simultaneous knockdown of both PKACα and PKACβ dramatically potentiated SeV-triggered induction of downstream genes. These results suggest that PKACα and PKACβ play redundant roles in inhibiting innate antiviral response, which is consistent with their redundant roles in regulation of many other cellular processes.

Our experiments suggest that PKACs inhibit innate antiviral response by targeting the central adaptor protein VISA, which is mostly localized at the mitochondria. Consistent with previous reports [35], we found that a fraction of PKACs was located in the mitochondria, and associated with VISA constitutively before and after viral infection. Overexpression of PKACs phosphorylated VISA at T54 and caused its degradation, whereas knockdown of PKACs inhibited SeV-induced phosphorylation of VISA at T54 and up-regulated its protein level. These results suggest that PKACs negatively regulate SeV-triggered induction of downstream genes by phosphorylating VISA at T54. Consistently, reconstitution experiments indicated that mutation of T54 of VISA to D, which mimics its phosphorylated status, abolished VISA activity, and this mutant failed to mediate SeV-triggered signaling and induction of downstream antiviral genes. Un-expected, mutation of T54 of VISA to A, which mimics its un-phosphorylated status, partially inhibited its activity. The exact reasons responsible for this observation is currently unknown. The simplest explanation is that mutation of T54 of VISA to A causes its conformational changes that partially affect its activity. In fact, there are numerous cases that mutation of T to A or D does not act in an opposite way. For examples, both S366A and S366D mutants of STING/MITA, or both S527A and S527D mutants of TBK1 have greatly reduced ability to mediate IFN-β induction [16, 36].

Our experiments suggest that phosphorylation of VISA by PKACs causes its inactivation by at least two processes. Firstly, the self-association, as well as SeV-induced aggregation of VISA(T54D), was decreased in comparison to wild-type VISA or VISA(T54A), whereas knockdown of PKACs increased VISA aggregation. Since virus-induced aggregation of VISA is an essential event for its activation, PKACs may inhibit virus-triggered signaling by phosphorylating VISA and impairing its aggregation. Secondly, phosphorylation of VISA by PKACs increased its K48- but not K63-linked polyubiquitination, whereas knockdown of PKACs decreased SeV-induced K48-linked polyubiquitination of VISA. In addition, SeV-induced K48-linked polyubiquitination and degradation of VISA(T54A) were abolished in comparison to wild-type VISA. These results suggest that phosphorylation of VISA at T54 by PKACs primes it for K48-linked polyubiquitination and degradation. We further showed that the E3 ubiquitin ligase MARCH5 but not RNF5 or AIP4 was responsible for mediating PKACs-primed K48-linked polyubiquitination of VISA. This is consistent with previous reports that MARCH5 is a mitochondrial-associated E3 ligase that negatively regulates virus-triggered induction of downstream genes at the late phase of infection [33]. It is possible that multiple E3 ligases are involved in regulation of VISA-mediated signaling in distinct cellular compartments and/or different phases of viral infection.

Previously, the involvement of PKA in the regulation of innate immune response has not been reported. We found that SeV infection caused decrease of cAMP levels at the early phase of infection, but induced increase of cAMP levels at the late phase of infection, which was correlated to the increased PKA activity at the late phase of infection. Since virus-induced increase of cAMP levels at the late phase of infection was abrogated in *Rig-I*^-/-^ cells, we conclude that virus-triggered induction of cAMP and PKA activity is dependent on RIG-I-mediated pathways. It has been well established that binding of a ligand to a G-protein coupled receptor (GPCR) activates adenylyl cyclase (AC), which catalyzes the synthesis of cAMP from ATP. It is possible that the RIG-I pathways directly or indirectly through GPCR activate an AC, which leads to induction of cAMP. In light of the observation that PKACs are constitutively associated with VISA in the mitochondria, our results suggest that virus-triggered induction of cAMP modulates VISA activity in a temporal manner. In un-infected cells, basal cAMP maintains VISA activity at a steady level. At the early phase of infection, cAMP levels are down-regulated, which decreases PKACs activity and promotes VISA activity for efficient induction of downstream antiviral genes. At the late phase of infection, cAMP levels and PKACs activity are increased, which inactivates VISA and attenuates innate antiviral response. Therefore, PKA attenuates innate antiviral response in a feed-back negative regulatory manner.

Our experiments suggest that PKA is not only important for negative regulation of innate antiviral response in cells, it is also essential for attenuating innate antiviral response in mice. We found that inhibition of PKA by the specific inhibitor H89 markedly potentiated SeV- and EMCV-induced expression of type I IFNs and IL-6 in the sera, and potentiated EMCV-induced death of infected mice. Because *Prkaca* and *Prkacb* double knockout is lethal in mice [37], we are currently unable to directly determine the effects of PKACα and PKACβ deficiency on innate antiviral response in animals. Nevertheless, our studies provide solid evidences for the feed-back negative regulation of VISA-mediated innate antiviral response by the house-keeping kinase PKA, and certainly help to understand how innate immune response is terminated at the late phase of viral infection to avoid host damage.

## Materials and methods

### Ethics statement

All animal experiments were performed in accordance with the Wuhan University animal care and use committee guidelines.

### Reagents and antibodies

Lipofectamine 2000 (Invitrogen); RNase inhibitor (Thermo); SYBR (Bio-Rad); mouse monoclonal antibodies against Flag, HA, and β-actin (Sigma), TBK1, phospho-TBK1 (Ser172) and phospho-Ser/Thr (Abcam), phospho-PKA substrate and phospho-IRF3 (Ser396) (Cell Signaling Technology), rabbit polyclonal antibodies against IRF3 (Santa Cruz Biotechnology) and rabbit polyclonal antibodies against VISA (Bethyl) were purchased from the indicated manufacturers; mouse anti- PKACα/β antisera were raised against recombinant human full-length PKACβ; SeV, EMCV and HSV-1 were previously described [38, 39]; HEK293 and THP1 cells (ATCC) were purchased from the indicated manufactures; HEK293T cells were originally provided by Dr. Gary Johnson (National Jewish Health).

### Constructs

NF-κB, ISRE, STAT1/2, IFN-β promotor, and IRF1 promoter luciferase reporter plasmids, mammalian expression plasmids for Flag- or HA-tagged RIG-I, MDA5, VISA, MITA, TBK1 and IRF3 were previously described [40–42]. Flag- or HA-tagged PKACα, PKACβ, PKACγ and their mutants were constructed by standard molecular biology techniques.

### RNAi

Double-strand oligonucleotides corresponding to the target sequences were cloned into the pSuper.retro RNAi plasmid (Oligoengine). The targeting sequences are as following. Human PKACα: 5′-GGGTGATGCTGGTGAAACA-3′; Human PKACβ: 5′-GAAGAGTCATGTTGGTAAA-3′. The targeting sequences for both human PKACα and PKACβ (PKACs): #1: 5′-GAAGGTTCAGTGAGCCCCA-3′; #2: 5′-TAGCCAAAGCCAAAGAAGA-3′. siRNA oligonucleotides sequence for both mouse PKACα and PKACβ (PKACs): 5′-TAGCCAAAGCCAAAGAAGATT-3′.

### Transfection and reporter assays

Cells (1×10^5^) were seeded in 24-well plates and transfected the following day by standard calcium phosphate precipitation. In the same experiment, empty control plasmid was added to ensure that each transfection receives the same amount of total DNA. To normalize for transfection efficiency, 0.01 μg of pRL-TK or pRL-SV40 (*Renilla* luciferase) reporter plasmid was added to each transfection [43, 44]. Luciferase assays were performed using a dual-specific luciferase assay kit (Promega).

### Co-immunoprecipitation and immunoblot analysis

For co-immunoprecipitation, cells (1×10^7^) were lysed in 1 mL NP-40 lysis buffer (20 mM Tris-HCl [pH 7.4], 150 mM NaCl, 1 mM EDTA, 1% Nonidet P-40, 10 μg/mL aprotinin, 10 μg/mL leupeptin, and 1 mM phenylmethylsulfonyl fluoride). For direct analysis of protein expression, cells were lysed with SDS-PAGE loading buffer followed by ultra-sonication. Co-immunoprecipitation and immunoblot analysis were performed as previously described [45, 46].

### Semi-denaturing detergent agarose gel electrophoresis (SDD-AGE)

SDD-AGE was performed as previously described [47].

### Ubiquitination assays

Ubiquitination assays were performed as previously described [48, 49].

### Statistics

GraphPad Prism software was used for all statistical analyses. Quantitative data displayed as histograms are expressed as means ± SD (represented as error bars). Data were analyzed using a Student’s unpaired t test or multiple t test. The number of asterisks represents the degree of significance with respect to p values. Statistical significance was set at a p < 0.05. Mice in each sample group were selected randomly in mouse experiments. The sample size (n) of each experimental group is described in the figure legend.
